# Prognostic Nomogram Associated with Longer Survival in Amyotrophic Lateral Sclerosis Patients

**DOI:** 10.14336/AD.2017.1016

**Published:** 2018-12-04

**Authors:** Qian-Qian Wei, Yongping Chen, Xueping Chen, Bei Cao, RuWei Ou, Lingyu Zhang, Yanbing Hou, Huifang Shang

**Affiliations:** Department of Neurology, West China Hospital, Sichuan University, Chengdu, Sichuan, China; Department of Neurology, West China Hospital, Sichuan University, Chengdu, Sichuan, China; Department of Neurology, West China Hospital, Sichuan University, Chengdu, Sichuan, China; Department of Neurology, West China Hospital, Sichuan University, Chengdu, Sichuan, China; Department of Neurology, West China Hospital, Sichuan University, Chengdu, Sichuan, China; Department of Neurology, West China Hospital, Sichuan University, Chengdu, Sichuan, China; Department of Neurology, West China Hospital, Sichuan University, Chengdu, Sichuan, China; Department of Neurology, West China Hospital, Sichuan University, Chengdu, Sichuan, China

**Keywords:** Amyotrophic lateral sclerosis, biomarkers, HbA1c, CK, creatinine, Nomogram

## Abstract

Better understanding of survival factors in amyotrophic lateral sclerosis (ALS) could help physicians and patients schedule therapeutic interventions. We conducted a study to evaluate the predictive factors associated with longer survival and construct prognostic nomogram in ALS patients. A total of 553 ALS patients were enrolled and divided into 2 groups: a training set and a validation set. Risk factors for survival were identified using logistic regression analysis, and a nomogram created by R program was performed to predict the probability of longer survival in the training set; then receiver operating characteristic (ROC) analysis was applied to assess predictive value of the nomogram model. The median survival time was 3.2 years for all patients. Multivariate analyses revealed that age of onset, rate of disease progression, hemoglobin A1c (HbA1c) level, body mass index, creatinine, creatine kinase (CK), and non-invasive positive pressure ventilation (NIPPV) were independent predictors of longer survival. A nomogram based on the above seven predictive factors was developed to predict the possibility of longer survival. The ROC curve of the nomogram demonstrated good discrimination ability with an AUC of 0.92 (95% CI: 0.88-0.96) in the validation set. In ALS, serum CK, creatinine and HbA1c levels at baseline were independent biomarkers of longer survival. The prognostic nomogram model that integrated all significant independent factors for those who survived longer than 3 years provides an effective way to predict the probability of longer survival and can help doctors evaluate the disease progression and give personalized treatment recommendations.

Amyotrophic lateral sclerosis (ALS) is a fatal neurodegenerative disease characterized by progressive degeneration of both upper and lower motor neurons in the brain and spinal cord [1]. Accompanied by respiratory failure, the death usually occurs in 3 to 5 years from the symptom onset, and approximately 5-10% of ALS patients have had survival times longer than 10 years [2]. Our previous study also found that the probabilities of survival from symptom onset were 58.4% at 3 years, 26.0% at 5 years, and 1.4% for longer than 10 years [3].

ALS has a considerable variability in outcome. A number of disease-related factors have previously been considered to be associated with survival in ALS. For example, younger age of onset, longer diagnostic delay, slower rate of disease progression, better cognitive function, and some genetic mutations and clinical phenotypes were positively correlated with longer survival [2, 4-6]. Therapeutic factors including taking riluzole, gastrostomy percutaneous endoscopy (PEG), tracheostomy, non-invasive positive pressure ventilation (NIPPV), and comprehensive care were related to longer survival [7]. There are contradictory findings on the relationship between sex, the site of onset, the El Escorial Criteria (EEC) category, the ALS functional rating scale-revised (ALSFRS-R) score and survival time [2, 8]. In addition, various biological markers have been proposed as potential predictors for survival with ALS. For example, studies have demonstrated that dyslipidemia [9], high body mass index (BMI), slow weight loss, elevated levels of uric acid, serum albumin, creatinine and creatine kinase (CK) [10-12] are significantly associated with better outcomes in ALS. There are no consistent conclusions on the relationship between ALS survival and personal comorbidities, such as a history of hypertension or diabetes mellitus (DM) [13, 14]. However, most of the previous studies were performed in small referral-based series and have not been confirmed by subsequent studies.

The prognostic factors for ALS are not satisfactorily defined in studies because of variable sample size, the selection criteria and the disease heterogeneity [2]. Better understanding of prognostic factors for ALS could help physicians and patients schedule therapeutic interventions. Therefore, risk stratification for ALS patients based on baseline features is an area of research interest. Most studies performed Cox proportional hazards analysis or Kaplan-Meier curves to identify prognostic predictors for survival in ALS [3, 11, 15, 16]. A few studies analyzed the correlation between several clinical markers evaluated at registration and survival time in ALS using logistic regression [17].

A nomogram was proposed as a novel tool to measure survival in the majority of cancer types [18]. It provides a graphical representation of the factors that can be applied to calculate the probability of survival for an individual patient by the points associated with each associated factor. There is no predictive nomogram analyzing the clinical features and serum markers for longer survival with ALS. This lack of a predictive nomogram prompted us to conduct a study to compare the clinical factors, personal history, comorbidities, several potential prognostic related hematological markers and treatments between ALS patients with longer survival times of beyond 3 years and those with survival times less than 3 years (according to the median survival time of our patients) and to reveal the factors associated with longer survival. We constructed a nomogram based on these independent factors for ALS patients to predict longer survival.

## MATERIALS AND METHODS

### Patients and follow-up

Patients who were diagnosed with definite or probable ALS by our neurologists according to the EEC registered in our tertiary referral center of southwest China (Department of Neurology, West China Hospital of Sichuan University, Chengdu, Sichuan province) from March 2009 to March 2014 (referred to as “baseline” in the current manuscript). Patients with progressive muscular atrophy, progressive bulbar paralysis, primary lateral sclerosis, frontotemporal dementia and juvenile ALS were excluded in the current study. Patients with additional diagnoses of other neurodegenerative diseases were also excluded. Follow-ups with patients were conducted by neurologists using a telephone or face-to-face interview in 3- or 6-month intervals (QQW, LYZ, YBH, RH, ZZZ). Death information was collected from provincial public security bureau records or family reports. All cases were followed until January 2017. Finally, a total of 553 patients who met the inclusion criteria with complete hematological data were enrolled in the study. Among these patients, 387 enrolled from 2009 to 2012 were used as the training set, while 166 patients from 2013 to 2014 were regarded as the validation set.

### Definition of clinical features and comorbidities

Information on the demographic features, personal history (cigarette smoking, drinking, occupation, pesticide exposure, and living environment were obtained through questionnaires as previously described [19]), and disease-related data, including age of onset, the region of symptom onset (upper limb, lower limb or bulbar), disease duration at baseline, diagnostic delay, ALSFRS-R score at baseline, dominant involvement of upper motor neurons (UMN-ALS), EEC and family history were collected at the patient’s first registration in our database.The ALSFRS-R includes three subscales: bulbar function (ALSFRS-R-B), motor function (ALSFRS-R-M) and respiratory function (ALSFRS-R-R). The rate of disease progression was assessed by the changes of ALSFRS-R per month (Formula: (48 - ALSFRS-R score at the baseline visit)/month intervals between first symptom onset and the baseline visit). Body mass index (BMI) values were categorized according to the World Health Organization (WHO) classification. Blood pressure was measured at the baseline, and hypertension was defined as systolic blood pressure ≥140 mmHg or diastolic ≥90 mmHg or the use of antihypertensive medications in the past 2 weeks. Participants were identified as having DM if they had a fasting blood glucose ≥7.0 mmol/L or were treated with insulin or oral hypoglycemic agents[20]. Hyperlipidemia was defined as the presence of any of the following: a history of hyperlipidemia, taking cholesterol-lowering drugs or fasting serum total cholesterol (TC) level ≥5.17 mmol/L or triglycerides (TG) ≥1.7 mmol/L. Depression and anxiety symptoms of patients were assessed using Hamilton Depression Rating Scale-24 items and Hamilton Anxiety Rating Scale. The mini-mental state examination was used to evaluate global cognition of the patients.

### Assessment of hematological markers

Blood samples after an overnight fasting (>8 hr) were collected at baseline. The following hematological tests were conducted in this study: hemoglobin A1c (HbA1c), hemoglobin, erythrocytes, total leukocytes, neutrophils, lymphocytes, thrombocytes, thyrotrophin, serum free triiodothyronine, free thyroxine, TC, TG, high-density lipoprotein (HDL), low-density lipoprotein (LDL), CK, creatinine, uric acid, urea, total bilirubin, total protein (TP), alanine aminotransferase, aspartate amino-transferase, and albumin. HbA1c levels were assessed by ion exchange high-pressure liquid chromatography (HPLC) on a Tosoh G7 standard mode (Tosoh Corporation, Japan) using reagents according to the manufacturer's instructions in the clinical laboratory. Biochemical indexes were measured by an enzymatic colorimetric method using an automatic analyzer (Olympus AU400; Olympus, Japan). The WHO criteria for anemia are hemoglobin levels less than 12 g/dL in premenopausal women and less than 13 g/dL in men and postmenopausal women [21]. Hypoproteinemia was defined as a TP level <60 g/L. Participants were categorized into groups based on their levels, and the lowest group was used as the reference.

The ALSFRS-R score, progression of symptoms, and treatments, as well as newly developed comorbidities or additional medications, were recorded at each follow up. The survival time for patients was defined as the interval time between date of onset and date of death or tracheotomy, which was taken as equivalent to death. The West China Hospital Research Ethics Committee approved this study. All participants signed written informed consents prior to participating in the study. All methods were performed in accordance with the relevant guidelines and regulations.

### Statistical analysis and nomogram construction

Continuous variables were presented as the mean ± standard deviation (SD). We divided the ALS patients into patients with survival times of beyond 3 years and less than 3 years. The comparisons of continuous variables were made using the Student’s t test or Mann-Whitney U test. A Chi-Square test (or Fisher’s exact test when appropriate) was implemented to analyze the categorical variables. Continuous variables were categorized into adequate forms to fit the proportional hazards. Associated factors with longer survival were studied using a binary logistic regression analysis. A nomogram was formulated based on the results of a multivariate analysis. The predictive accuracy of the nomogram was then graphically displayed using receiver operating characteristic (ROC) and quantified by the area under the curve (AUC). An AUC of 1.0 indicates a perfect concordance, whereas an AUC of 0.5 shows no relationship [22]. Furthermore, the nomogram was subjected to 1000 bootstrap resamples for reduction of overfit bias and for internal validation with logistic calibration plot. Bootstrapping allows for the simulation of the performance of the nomogram if applied to future patients and provides an estimate of the average optimism of the AUC. All analyses and graphics were performed with SPSS 19.0 (SPSS, Inc., Chicago, IL, USA) and R (version 3.1.2; www.R-project.org/). All statistical tests were two-tailed, and P <0.05 was considered statistically significant.

**Table 1:** Demographic and disease-related characteristics of the ALS patients related to survival time. N (%)

Factors	Training set				Validation set				P*
	Event 3 (-) N = 178	Event 3 (+) N = 209	Total N = 387	P	Event 3 (-) N = 70	Event 3 (+) N =96	Total N = 166	P	
Age of onset, years				0.001*				0.004*	0.287
<40	14 (7.9)	34 (16.3)	48 (12.4)		5 (5.1)	24 (25.0)	29 (17.5)		
40-45	15 (8.4)	36 (17.2)	51 (13.2)		4 (5.7)	14 (14.6)	18 (10.8)		
45-50	16 (9.0)	21 (10.0)	37 (9.6)		3 (4.3)	5 (5.2)	8 (4.8)		
50-55	12 (6.7)	15 (7.2)	27 (7.0)		8 (11.4)	9 (9.4)	17 (10.2)		
55-60	36 (20.2)	45 (21.5)	81 (20.9)		13 (18.6)	18 (18.8)	31 (18.7)		
60-65	35 (19.7)	32 (15.3)	67 (17.3)		16 (22.9)	15 (15.6)	31 (18.7)		
65-70	22 (12.4)	14 (6.7)	36 (9.3)		10 (14.3)	8 (8.3)	18 (10.8)		
>70	28 (15.7)	12 (5.7)	40 (10.3)		11 (15.7)	3 (3.1)	14 (8.4)		
ALSFRS-R				0.003*				0.002*	0.399
≥40	76 (42.7)	121 (57.9)	197 (50.9)		23 (32.9)	55 (57.3)	78 (47.0)		
<40	102 (57.3)	88 (42.1)	190 (49.1)		47 (67.1)	41 (42.7)	88 (53.0)		
Disease duration				<0.001*				<0.001*	0.355
≥12 months	61 (34.3)	151 (72.2)	212 (54.8)		29 (41.4)	69 (71.9)	98 (59.0)		
<12 months	117 (65.7)	58 (27.8)	175 (45.2)		41 (58.6)	27 (28.1)	68 (41.0)		
Disease delay				<0.001*				0.001*	0.285
≥12 months	57 (32.0)	143 (68.4)	200 (51.7)		29 (41.4)	65 (67.7)	94 (56.6)		
<12 months	121 (68.0)	66 (31.6)	187 (48.3)		41 (58.6)	31 (32.3)	72 (43.4)		
Disease progression				<0.001*				<0.001*	0.991
<0.5	30 (16.9)	121 (57.9)	151 (39.0)		8 (11.4)	53 (55.2)	61 (36.7)		
0.5-1.0	66 (37.1)	71 (34.0)	137 (35.4)		26 (37.1)	33 (34.4)	59 (35.5)		
1.0-1.5	32 (18.0)	13 (6.2)	45 (11.6)		14 (20.0)	7 (7.3)	21 (12.7)		
1.5-2.0	25 (14.0)	4 (1.9)	29 (7.5)		10 (14.3)	3 (3.1)	13 (7.8)		
2.0-2.5	10 (5.6)	0 (0.0)	10 (2.6)		4 (5.7)	0 (0.0)	4 (2.4)		
>2.5	15 (8.4)	0 (0.0)	15 (3.9)		8 (11.4)	0 (0.0)	8 (4.8)		
HbA1c				<0.001*				<0.001*	0.994
<5.0	16 (9.0)	19 (9.1)	35 (9.0)		4 (5.7)	10 (10.4)	14 (8.4)		
5.0-5.5	54 (30.3)	107 (51.2)	161 (41.6)		18 (25.7)	54 (56.2)	72 (43.4)		
5.5-6.0	58 (32.6)	55 (26.3)	113 (29.2)		23 (32.9)	25 (26.0)	48 (28.9)		
6.0-6.5	30 (16.9)	20 (9.6)	50 (12.9)		14 (20.0)	6 (6.2)	20 (12.0)		
6.5-7.0	10 (5.6)	4 (1.9)	14 (3.6)		5 (7.1)	0 (0.0)	5 (3.0)		
>7.0	10 (5.6)	4 (1.9)	14 (3.6)		6 (8.6)	1 (1.0)	7 (4.2)		
BMI				<0.001*				<0.001*	0.088
<18	14 (7.9)	11 (5.3)	25 (6.5)		5 (7.1)	5 (5.2)	10 (6.0)		
18-20	53 (29.8)	25 (12.0)	78 (20.2)		17 (24.3)	7 (7.3)	24 (14.5)		
20-22	65 (36.5)	29 (13.9)	94 (24.3)		35 (50.0)	12 (12.5)	47 (28.3)		
22-24	16 (9.0)	73 (34.9)	89 (23.0)		4 (5.7)	37 (38.5)	41 (24.7)		
24-26	21 (11.8)	45 (21.5)	66 (17.1)		4 (5.7)	21 (21.9)	25 (15.1)		
26-28	1 (0.6)	2 (1.0)	3 (8.3)		0 (0.0)	7 (7.3)	7 (4.2)		
>28	8 (4.5)	24 (11.5)	32 (8.3)		5 (7.1)	7 (7.3)	12 (7.2)		
Creatinine				0.051				0.057	0.879
<40	17 (9.6)	9 (4.3)	26 (6.7)		7 (10.0)	3 (3.1)	10 (6.0)		
40-60	81 (45.5)	84 (40.2)	165 (42.6)		31 (44.3)	36 (37.5)	67 (40.4)		
60-80	59 (33.1)	93 (44.5)	152 (39.3)		25 (35.7)	35 (36.5)	60 (37.7)		
>80	21 (11.8)	23 (11.0)	44 (11.4)		7 (10.0)	22 (22.9)	22 (22.9)		
CK				0.058				0.003*	0.592
<50	22 (12.4)	15 (7.2)	37 (9.6)		8 (11.4)	6 (6.2)	14 (8.4)		
50-100	54 (30.3)	53 (25.4)	107 (27.6)		26 (37.1)	20 (20.8)	46 (27.7)		
100-240	78 (43.8)	95 (45.5)	173 (44.7)		29 (41.4)	39 (42.0)	68 (41.0)		
>240	24 (13.5)	46 (22.0)	70 (18.1)		7 (10.0)	32 (32.3)	38 (22.9)		
Uric acid				0.073				0.055	0.352
<220	29 (16.3)	39 (18.7)	68 (17.6)		8 (11.4)	12 (12.5)	20 (12.0)		
220-300	71 (39.9)	65 (31.1)	136 (35.1)		34 (48.6)	33 (34.4)	67 (40.4)		
300-380	52 (29.2)	55 (26.3)	107 (27.6)		20 (28.6)	24 (25.0)	44 (26.5)		
>380	26 (14.6)	50 (23.9)	76 (19.6)		8 (11.4)	27 (28.1)	35 (21.1)		
Total bilirubin				0.034*				0.001*	0.730
<9.0	41 (23.0)	54 (25.8)	95 (24.5)		17 (24.3)	24 (25.0)	41 (24.7)		
9.0-11.5	54 (30.3)	48 (23.0)	102 (26.4)		21 (30.0)	19 (19.8)	40 (24.1)		
11.5-15.0	49 (27.5)	44 (21.1)	93 (24.0)		26 (37.1)	21 (21.9)	47 (28.3)		
>15.0	34 (19.1)	63 (30.1)	97 (25.1)		6 (8.6)	32 (33.3)	38 (22.9)		
TP				0.031*				0.094	0.194
<60	25 (14.0)	15 (7.2)	40 (10.3)		9 (12.9)	4 (4.2)	13 (7.8)		
60-65	44 (24.7)	74 (35.4)	118 (30.5)		18 (25.7)	28 (29.2)	46 (27.7)		
65-70	63 (35.4)	63 (30.1)	126 (32.6)		23 (32.9)	25 (26.0)	48 (28.9)		
>70	46 (25.8)	57 (27.3)	103 (26.6)		20 (28.6)	39 (40.6)	59 (35.5)		
Hypoproteinemia				0.027*				0.024*	0.803
Yes	25 (14.0)	15 (7.2)	40 (10.3)		11 (15.7)	5 (5.2)	16 (9.6)		
No	153 (86.0)	194 (92.8)	347 (89.7)		59 (84.3)	91 (94.8)	150 (90.4)		
NIPPV				0.048*				0.044*	0.002*
Yes	9 (5.1)	22 (10.5)	31 (8.0)		7 (10.0)	21 (21.9)	28 (16.9)		
No	169 (94.9)	187 (89.5)	356 (92.0)		63 (90.0)	75 (78.1)	138 (83.1)		

Abbreviations: Event 3 (+) group = Survival time of beyond 3 years; Event 3 (-) group = Survival time of less than 3 years; N = Number; ALSFRS-R = Amyotrophic lateral sclerosis functional rating scale-revised; HbA1c = Hemoglobin A1c; BMI = body mass index; CK = Creatine kinase; TP = Total protein; NIPPV = Noninvasive positive pressure ventilation.

* Significant difference

P*: the difference between the training set and the validation set.

**Table 2:** Logistic regression analysis of the associated factors of longer survival (beyond 3 years) in ALS patients.

Factors	Univariate analysis		Multivariate analysis	
	OR (95%CI)	P-value	OR (95%CI)	P-value
Age of onset	0.793 (0.720-0.873)	<0.001	0.816 (0.723-0.920)	0.001
Disease progression	0.310 (0.234-0.410)	<0.001	0.293 (0.214-0.400)	<0.001
HbA1c	0.773 (0.646-0.925)	0.005	0.792 (0.631-0.993)	0.044
BMI	1.574 (1.353-1.831)	<0.001	1.565 (1.297-1.888)	<0.001
Creatinine	1.282 (0.989-1.662)	0.060	1.470 (1.041-2.075)	0.029
CK	1.376 (1.088-1.739)	0.008	1.443 (1.065-1.956)	0.018
Uric	1.144 (0.936-1.400)	0.189	-	-
Total bilirubin	1.109 (0.926-1.327)	0.260	-	-
TP	1.050 (0.853-1.292)	0.648	-	-
Hypoproteinemia	0.473 (0.241-0.929)	0.030	-	-
NIPPV	2.209 (0.990-4.931)	0.053	4.094 (1.300-12.891)	0.016

Abbreviations: ALS = Amyotrophic lateral sclerosis; OR = Odds ratio; CI = Confidence interval; HbA1c = Hemoglobin A1c; BMI = body mass index; CK = Creatine kinase; TP = Total protein; NIPPV = Noninvasive positive pressure ventilation.

## RESULTS

A total of 553 patients were enrolled in this study, including 305 male and 248 female patients. The mean age of onset was 55.9±12.8 years old (ranging from 25 to 85 years). The median survival time was 3.2 years for all patients (ranging from years 0.8 to 10.6 years). Three hundred and eighty-seven patients, with 209 patients in the Event 3 (+) group (survival time of beyond 3 years) and 178 patients in the Event 3 (-) group (survival time of less than 3 years), were enrolled in the training set. One hundred and sixty-six patients, with 96 patients in the Event 3 (+) group and 70 patients in the Event 3 (-) group, were enrolled in the validation set. There were no significant differences between the training and validation sets in the demographic, clinical and hematological variables (all with a p^*^ value >0.5) except for the NIPPV treatment, indicating a similar constitution and a balanced baseline between the two groups.

The clinical characteristics of patients in the training and validation sets are listed in Table 1. Younger age of onset, higher ALSFRS-R score at baseline, longer disease duration at baseline, longer diagnostic delay, lower rate of disease progression, lower HbA1c level, higher BMI, higher total bilirubin, lower proportion with hypoproteinemia, and higher proportion of NIPPV treatment were observed in the Event 3 (+) group versus the Event 3 (-) group (Table 1). Variables with p-values less than 0.1 are also shown in Table 1, including creatinine, CK, uric acid, and TP. No differences in the other parameters were found between the Event 3 (+) and Event 3 (-) groups (Supplement Table 1). As reported in Table 2, logistic regression analysis was calculated to determine the associated factors for the Event 3 (+) group. In the univariate analysis, Event 3 (+) was associated with clinical and hematological factors, such as age of onset, disease progression, HbA1c level, BMI, CK, and hypoproteinemia. Multivariate analyses revealed that age of onset (OR=0.816, P=0.001), rate of disease progression (OR=0.293, P<0.001), HbA1c level (OR=0.792, P=0.044), BMI (OR=1.565, P<0.001), creatinine (OR=1.470, P=0.029), CK (OR=1.443, P=0.018), and NIPPV (OR=4.094, P=0.016) were independent predictors of the Event 3 (+) group; no other hematological factors were retained in the model (Table 2).

**Figure 1.**
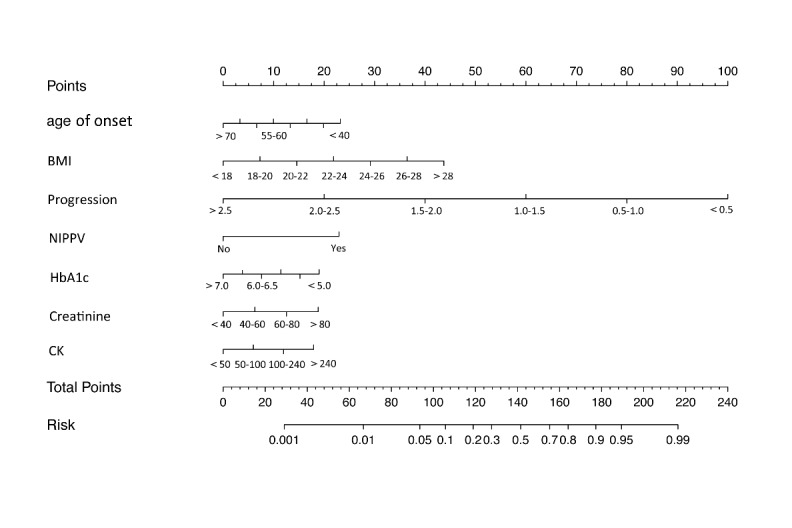
A nomogram composed of all independent factors to predict the probability of longer survival in ALS patients. The probability of longer survival in ALS is calculated by drawing a line to the point on the axis for each of the following variables: age, BMI, rate of disease progression, HbA1c, CK, creatinine, and NIPPV. The points for each variable are summed and located on the total point line. Next, a vertical line is projected from the total point line to the predicted probability bottom scale to obtain the individual probability of longer survival.

**Figure 2.**
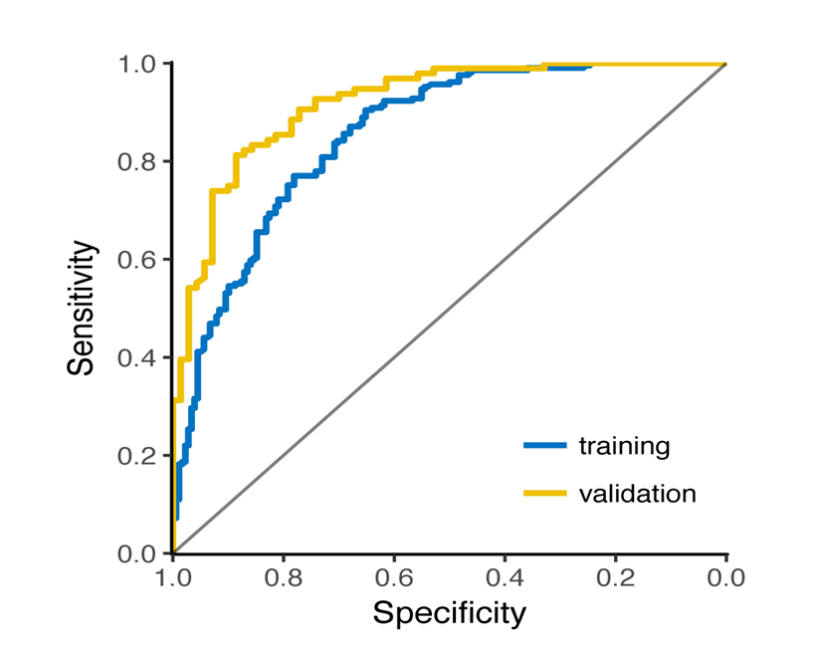
Predictive accuracy of the nomogram model. A receiver operating characteristics (ROC) curve of the multivariate logistic regression model for predicting longer survival in ALS patients, which had an AUC of 0.92 (95% CI: 0.88-0.96) in the validation set, implying a good concordance and a reliable ability.

The prognostic nomogram model that integrated all significant independent factors for the Event 3 (+) group in the training set is shown in Figure 1. For each patient, points were assigned for each of these independent factors (age of onset, rate of disease progression, HbA1c, BMI, creatinine, CK and NIPPV), and a total point, calculated from the nomogram, was visually indicated as a predictive probability for the Event 3 (+) group. Furthermore, ROC curve and a calibration plot are displayed to validate the predictive accuracy of the nomogram model. ROC in Figure 2 illustrated an AUC of 0.92 (95% CI: 0.88-0.96) in the validation set, which revealed a good concordance and a reliable ability.

## DISCUSSION

In the hospital-based study, we evaluated 553 ALS patients and revealed seven independent prognostic predictors for the survival of ALS, including age of onset, rate of disease progression, BMI, HbA1c, creatinine, CK, and NIPPV. Furthermore, a nomogram based on the above predictive factors was developed to predict the possibility of longer survival.

In this current study, we found that patients with younger age of onset had longer survival,and the rate of disease progression evaluated by ALSFRS-R decline at baseline was also associated with survival, which was supported by some previous studies [3, 4, 17, 23, 24]. Although bulbar onset was traditionally considered as an independent negative prognostic indicator [25], there was inconsistent finding. A multicenter prospective study on 451 sporadic ALS patients reported that upper limb weakness, lower limb weakness and bulbar symptoms had no significant impact on survival [26]. Another study based on an ALS cohort registered in the United States between 1999 and 2004 showed that bulbar onset was related to functional decline but survival time [27]. Conversely, previous study also found that the clinical form with lower limb onset was associated with a longer survival time than those with upper limb onset or bulbar onset. These inconsistent findings could be due to differences in study designs or patient selection criteria, or changes in ALS outcome as a result of effective treatment of dysphagia [28]. The current study found no impact of sex on survival in ALS, which was supported by our previous research and other studies [3, 12, 29]. In our study, bulbar-onset group had older age of onset than limb-onset group (mean age of onset: 59.4 years vs. 53.8 years, P = 0.001), and bulbar-onset group had higher proportion of female than limb-onset group (proportion of female: 57.8% vs. 42.1%, P = 0.011). Moreover, we further divided patients into three groups (bulbar onset, upper limb onset and lower limb onset). The univariate analysis showed that site of onset had no significant effect on longer survival (P trend=0.25, HR=1.19). The p value is more than 0.1. According to the design of multivariate analysis and Nomogram, the site of onset did not enter the model. Furthermore, our multivariate analysis revealed baseline BMI rather than hyperlipidemia to be an independent survival predictor, which was consistent with a previous study [10].

Recently, a study indicated that smoking predicts poor survival in ALS patients in a dose-response gradient [29]. Other studies also reported that a history of contact with pesticides and being from rural areas were associated with poorer survival [15, 17], while another study suggested the residence region and occupation at the index date had no effect on ALS survival [3]. In our study, personal history including smoking, drinking, living environment, pesticide exposure, and occupation seems to not have an impact on the survival of ALS patients. The complex relationship between personal history and survival remains to be clarified.

The findings of the association between hypertension and survival in ALS are inconsistent. Hypertension can cause oxygen deprivation through microangiopathy and reduce neural perfusion, thus exacerbating the degeneration of motor neurons and acting as a disease-modifying factor in ALS [30]. A study described hypertension as a protective factor for ALS [31], whereas another study found no association between hypertension and survival with ALS, that was consistent with our finding [32]. However, a study found that the severity of hypertension had a greater impact on survival with ALS than the duration of hypertension [30]. Therefore, more detailed data on hypertension should be collected and analyzed in a future study.

A previous study suggested that a history of pre-morbid DM2 is not an independent prognostic factor for ALS [33]. Consistently, fasting blood glucose (FBG) was not associated with survival in our cohort. However, our study suggested a negative relationship between baseline HbA1c levels and survival. We also observed a dose-response correlation between higher baseline HbA1c levels and higher future risks of mortality in ALS (unpublished). Compared with FBG, HbA1c levels are more sensitive because they indicate the average blood glucose levels during the previous 2 to 3 months; thus, HbA1c testing has been recommended as a standard of care for testing and monitoring DM [34]. Several potential mechanistic pathways have been indicated the association between HbA1c and ALS survival, including mitochondrial dysfunction, oxidative stress, and insulin resistance [35, 36].

**Figure 3.**
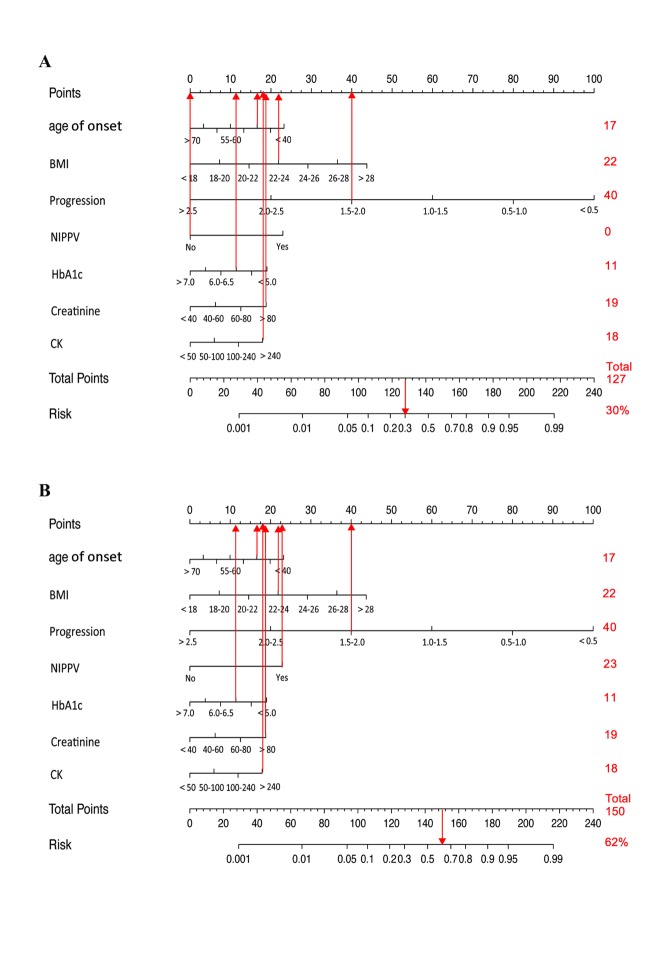
Examples for Nomogram. (A) Nomogram of 3 years survival for a patient with an onset age at 48, a BMI value of 23, a disease progression rate of 1.8, an HbA1c value of 5.6, a CK value of 260 IU/L, a creatinine value of 85 µmol/L, and who did not receive NIPPV. (B) Nomogram of 3 years survival for a patient with an onset age of 48, a BMI value of 23, a disease progression rate of 1.8, a HbA1c value of 5.6, a CK value of 260 IU/L, a creatinine value of 85 µmol/L, and who received NIPPV.

CK and creatinine can reflect muscular metabolism and have been investigated as biomarkers for ALS survival [11, 12]. Two studies with small sample sizes found no significant difference in survival in patients with elevated CK levels compared with those in the normal CK group [37, 38]. Another study consisting of 512 ALS patients noted that CK levels correlated positively with serum creatinine and seemed to be independent prognostic factors for survival [12]. A registry study with a cohort of 638 ALS patients showed that serum creatinine measured at diagnosis is an independent predictor for survival, because it reflects the state of fat-free muscle mass of the individual [11]. Serum creatinine has also been found to be a predictive factor for survival in patients with spinal and bulbar muscular atrophy [11]. Consistent with these studies, our study demonstrated a significant survival benefit in patients with higher CK and creatinine levels, after adjusting for other prognostic factors in multivariate analysis.

Riluzole is recommended treatment of ALS, which improves patients’ survival. In our study, the frequencies of treatment with Riluzole were no significant difference between ALS patients with survival longer and shorter than three years groups. The univariate analysis showed that treatment with Riluzole had no significant effect on the longer survival (P trend=0.12, HR=1.17). The p value is more than 0.1. First, the percentage of riluzole use in our cohort (178/553, 32.3%) was lower compared to western countries and Japan, possibly due to the economic cost and the concern about side effects. Second, it has been proved that riluzole can extend the survival time for several months. Since we set 3-years as the time point, the effect of riluzole on 3-year duration in our cohort may not appear since it could be influenced by the above two reasons. The percentage of other supporting therapies, including NIPPV and PEG, was also low, which may be due to the lack of comprehension of patients and their caregivers. NIPPV had positive effects on survival in the multivariate analysis, suggesting a better understanding of supportive management is necessary to improve survival with ALS. In China, if the patient does not have any symptoms of respiratory insufficiency, the patient and caregivers do not accept NIPPV treatment. However, if the patient has any respiratory problems, he was recommended NIPPV treatment and usually the patient will use NIPPV treatment if no economic problem. In our study, our patients who took NIPPV treatment only extend the timing spent in the stage 4 by using king’s college staging system because our patient using NIPPV at stage 4 (unpublished data).

It is reasonable that a diagnosis of definite ALS with widespread clinical involvement carries a significantly poorer prognosis than other diagnostic categories [15]. However, the EEC category at register was not a significant independent predictor of the outcome in the current study. This discrepancy could be explained by the small proportion of probable ALS (18.4%) at baseline enrollment in our cohort. For the clinical phenotypes of ALS, the proportions of UMN-D ALS were no significant difference between the two groups regarding survival time. Other phenotypes should be recorded to explore the effect on ALS survival in further study.

Nomograms have been developed in some cancers,and they were shown to be more accurate than the conventional staging systems for predicting prognoses [18]. Thus, a prognostic nomogram, corresponding to a predictive model including the independent factors associated with longer survival in ALS, was constructed in our study. An ROC curve and a calibration plot were applied to validate this nomogram, the results showed good predictive accuracy, concordance and reliability were identified with the use of our nomogram to estimate the survival of ALS patients. This nomogram offered an effective method to predict which kind of ALS patients may have likelihood of longer survival, and additional information for disease progression and more appropriate treatments could be provided to some ALS patients according to the nomogram. For example, a patient with an onset age at 48, with a BMI value of 23, a disease progression rate of 1.8, an HbA1c value of 5.6, a CK value of 260 IU/L and a creatinine value of 85 µmol/L has more than a 30% possibility of survival longer than 3 years without considering other factors (Figure 3a). However, if the patient has treatment with NIPPV, this nomogram predicts that the possibility of survival longer than 3 years increases by 32% (changing from 30% to 62%, Figure 3b). If the patient has a higher BMI at baseline, such as 27, he/she also has more than a 50% possibility of survival longer than 3 years even without the treatment of NIPPV. It has been mentioned that NIPPV provides a significant advantage to ALS patients in terms of median survival. The necessity of NIPPV in an early stage of disease is a negative survival factor. It may indicate the progression of the disease in the ALS patients with the necessity of NIPPV in an early stage of disease may be more severe. However, it remains uncertain whether use of NIPPV as early as possible can protect the disease progression longer survival even when patients do not reach the criteria of FVC, though one study found early NIPPV increased survival compared with late NIPPV[39]. BMI is a generally accepted marker of nutritional status, having a positive effect on survival. Increasing evidence implicates a causal role for metabolic dysfunction in ALS, suggesting that optimizing nutrition could prolong survival. Therefore, we believe this nomogram model will assist clinicians in formulating a treatment strategy and supportive management plan for ALS patients in terms of the probability of longer survival.

To our knowledge, this is the first study providing a nomogram to predict longer survival in ALS. The strength of this study is the analysis of a large sample size of ALS patients with detailed information on multiple parameters. However, this study also had limitations. The patients were registered from a single center and may not represent the Chinese ALS patients as a whole, so it needs further multicenter validation in China and external validation in different ethnic populations. Low proportion of patients with Riluzole treatment was reported in our patient sample, which may underestimate the effect of Riluzole. It also may lead to some bias in the onset date of disease when some patients who had longer disease duration at the first visit in our hospital. Some other parameters, such as causative gene mutations, forced vital capacity, and ferritin [25, 29, 40], which might also have an effect on survival were not investigated for all of the ALS patients at the time of recruitment.

### Conclusions

In this large center-based cohort, longer survival in ALS is associated with younger age, slower rate of disease duration, higher BMI, CK, creatinine at baseline, lower HbA1c levels, and NIPPV treatment, after correction for other known factors. The nomogram proposed an effective way to predict the likelihood of longer survival and may be helpful for individual ALS patient to obtain additional information about disease progression and to receive appropriate treatment. These findings also have relevant implications in the design of future clinical trials. Longitudinal collection of the variables of hematological factors of ALS patients from different ethic groups will help determine whether and how they vary during the progression of the disease.
